# 2298. Penetration of SARS-CoV-2 Alpha, Delta, and Omicron Variants in the USA

**DOI:** 10.1093/ofid/ofad500.1920

**Published:** 2023-11-27

**Authors:** Hosoon Choi, Munok Hwang, John David Coppin, Piyali Chatterjee, Chetan Jinadatha

**Affiliations:** Central Texas Veterans Health Care System, Temple, Texas; Central Texas Veterans Health Care System, Temple, Texas; Central Texas Veterans Health Care System, Temple, Texas; Central Texas Veterans Health Care System, Temple, Texas; Central Texas Veterans Health Care System, Temple, Texas

## Abstract

**Background:**

Since the beginning of the pandemic in 2019, SARS-CoV-2 virus has undergone numerous mutations and several variants of concern (VOCs) have emerged. Here, we investigated the transmission of SARS-CoV-2 based on the virus sequencing data from the world, USA, and the local area (Temple, TX). We examined the changes in prevalence of the WHO designated VOCs Alpha, Delta, and Omicron variants in the world, USA, and Temple, TX for past three years. In addition, the time interval until the emergence of these VOCs in 20 major cities of USA and the local area were compared.

**Methods:**

The metadata for weekly sequenced cases of VOCs Alpha, Delta, and Omicron for the world and USA were downloaded from GISAID. The local data consisted of 8154 SARS-CoV-2 samples collected from patients of Central Texas VA hospital located in Temple, TX from March 2020 to April 2023. Our local catchment includes rural and urban area. All SARS-CoV-2 positive samples were sequenced regardless of their Cycle Threshold (CT) values. The penetration of VOCs in the USA was determined by the days from the first detection of each variant anywhere in the world until the first date that each variant appeared in each city.

**Results:**

The prevalence changes of VOCs were similar across all three of our comparison areas (Figure 1A). While Alpha and Delta showed gradual increase, Omicron showed a sharp increase. The fold increase of cases between Alpha to Delta, Delta to Omicron, and Alpha to Omicron were much bigger for Temple, TX than the US or across the world (Figure 1C). The penetration of VOCs differed by region and by variants (Table 1 & Figure 2). Overall, the penetration of Alpha and Delta was much slower than Omicron. The penetration of Alpha to major cities took 41 to 419 days, and Delta penetration took 0 to 441 days. However, Omicron was penetrated to all major cities in only 12 to 42 days (Figure 2).

**Figure:**
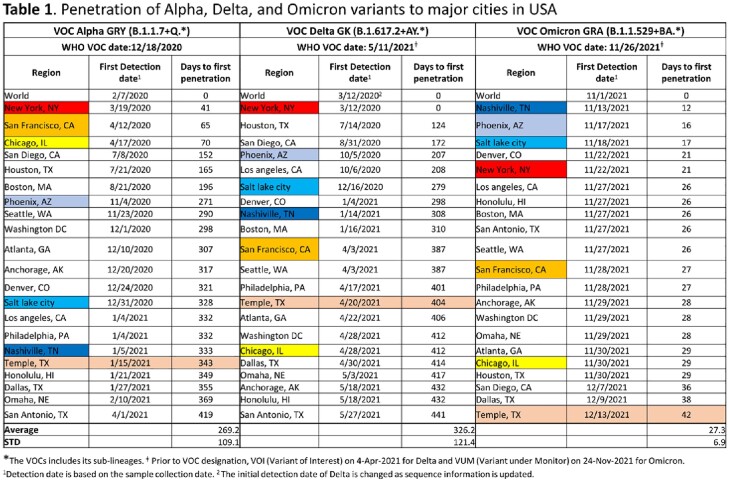

Penetration of the VOCs in the USA

Figure 1

**Figure:**
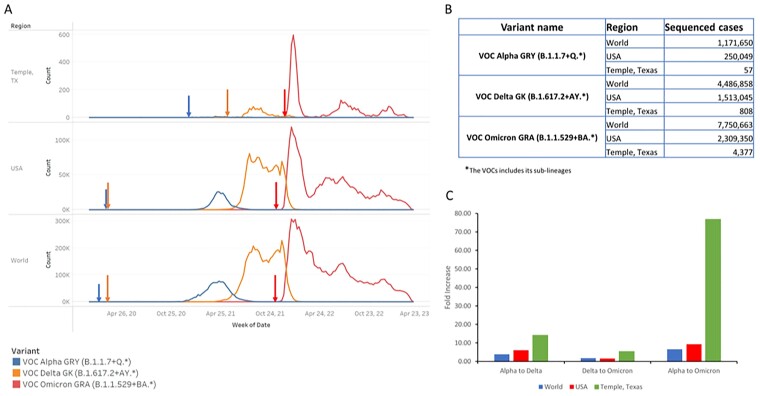

The changes in prevalence of the VOC variants Alpha, Delta, and Omicron in the world, USA and local catchment area and its impact. A. The number of VOCs in the world, USA and Temple, TX region. The number is compounded weekly from the week of March 15, 2020, to April 2, 2023. For the World and USA, data analysis is based on the samples sequenced and uploaded to GISAID database. Arrows indicate first detection date of each variant. B. The total number of sequenced cases for each VOCs in each region. C. The fold increase of the number of variants in the transition between Alpha to Delta, Delta to Omicron, and Alpha to Omicron in three regions.

**Conclusion:**

By analyzing regional penetration, our study demonstrates the diverse transmission characteristics of Alpha, Delta, and Omicron variants. While the penetration of new VOCs such as Alpha and Delta was slower during the early years of the pandemic, Omicron spread was much more rapid. Understanding these transmission patterns of SARS-CoV-2 can help public health entities and the federal government better plan initiatives that help protect their citizens.

Figure 2

**Figure:**
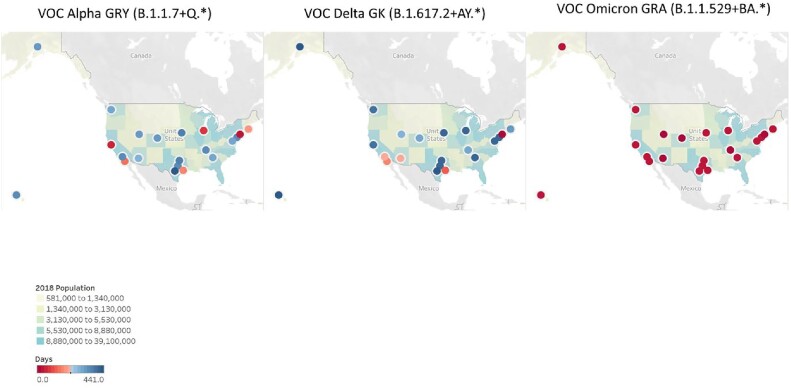

Penetration of the VOC variants to major cities in USA. Days were calculated from the first detection date of each variant in the world till its appearance in the city. The red gradients indicate early emergence while blue gradients indicate late arrival. Background map shows 2018 population of the areas.

**Disclosures:**

**All Authors**: No reported disclosures

